# Twitter as a Medical Media Among French Young Oncologists: Results from a National Survey

**DOI:** 10.1007/s13187-021-02119-7

**Published:** 2021-11-26

**Authors:** Matthieu Roulleaux Dugage, Natacha Naoun, Côme Bommier, Morgan Michalet, Yohann Loriot, Pierre Blanchard, Marc Hilmi, Jean-Charles Soria

**Affiliations:** 1 Department of Medical Oncology, Gustave Roussy, Université Paris Saclay, 114 rue Edouard Vaillant, 94800 Villejuif, France; 2 Association Pour L’Enseignement Et La Recherche Des Internes d’Oncologie, AERIO, Paris, France; 3 Hemato-Oncology Department, Assistance Publique Hôpitaux de Paris, Hôpital Saint Louis, Paris, France; 4 Association Des Internes en Hématologie, AIH, Paris, France; 5 Department of Radiation Oncology, Institut du Cancer de Montpellier, Montpellier, France; 6 SFJRO, Société Française Des Jeunes Oncologues Radiothérapeutes, Paris, France; 7 Department of Radiation Oncology, Gustave Roussy, Université Paris Saclay, Villejuif, France

**Keywords:** Residency, Young oncologists, Medical continuous education, Social media, Twitter

## Abstract

Since its launch in 2006, Twitter has become a commonly used platform for sharing medical information, especially in the field of oncology. However, its role and impact on young oncologists’ education remain unclear. Moreover, COVID-19 and congress virtualization is likely to have modified Twitter use by the medical society.

We conducted a national survey (27 questions) in France among medical oncology, hematology, and radiation therapy young doctors to help better understand the role played by Twitter on their medical education. One hundred eighty-three young oncologists participated in our survey. A majority does not use Twitter (72.1%), mostly to reduce their time spent on social media. Participants using Twitter (27.9%) often use it more than once a week, mostly by scrolling on their news feed. Interestingly, they rarely express their own opinion on Twitter: a majority of them (75.5%) tweet less than once a month while the rest of them mostly retweet others’ tweets. They mainly follow English-speaking experts, scientific societies, and medical journals. Pharmaceutical laboratories’ accounts are of less significance. Overall Twitter usage seems increasing since COVID-19 pandemic and the consequent digitalization of congresses. No statistical difference was observed between the baseline characteristics of Twitter users and non-users.

This survey shows that Twitter is a relevant mean of continuous medical education used by around a third of French young oncologists, especially since COVID-19 pandemic and the virtualization of congresses. This media should be considered and evaluated for its educational advantages or potential biases.

**Supplementary Information:**

The online version contains supplementary material available at 10.1007/s13187-021-02119-7.

## Introduction

For the last two decades, Internet has deeply modified our way to practice and learn medicine. Social networks have become ubiquitous, and with them, the conventional education means have been overturned. More and more students now use digital media for their formation, including medical education [1].

Twitter is a microblogging network that was created in 2006, where users can share contents within a 140-character limit. It has quickly grown into one of the most used social networks, with around 330 million users in 2019, around half of which declare a daily use [2]. In the medical field, Twitter is recognized as a useful platform for scientific discussion, literature monitoring (through dedicated accounts), or press releases [3]. It is a meeting point for medical doctors, scientists, and patients’ associations that allows an increase in the visibility of shared articles [4]. Because clinical research is central in oncology, it is probably one of the fields where Twitter is the most useful.

Recently, COVID-19 pandemic has pushed the international medical community to attend online congresses, and medical content has increased on the platform [5]. It is therefore relevant to ask whether COVID-19 has had an influence on Twitter use by the medical community.

Twitter has also become a customary tool for medical students: it seems complementary to academic courses [6, 7], and a few studies even suggest its educational benefit [8, 9]. However, the impact on their formation has not been well studied so far [10]. Similarly, despite an increasing literature on this subject [11], there is little evidence on how Twitter is used by young oncologists for their continuous education, and what advantage they find in it.

Moreover, even if the proportion of medical practitioners using this network seems rising, only a few express themselves, whereas a large majority retweet medical influencers’ content [12]. Younger doctors seem overrepresented on Twitter [13], and as in social media in general, representativeness is therefore in question [14].

We conducted a national survey in France to help better understand the role of Twitter in the continuous medical education of young oncologists and its possible evolution due to COVID-19 pandemic and congress digitalization.

## Materials and Methods

### Survey Description

We developed a questionnaire aiming at understanding the role of Twitter in the continuous formation of French young oncologists, which was validated by a working group comprised of resident and senior clinicians.

Twenty-seven questions were asked, concerning medical curriculum (4 questions), the use of Twitter (2 questions), the characterization of Twitter non-users (5 questions), the characterization of Twitter users (10 questions), and the possible evolution due to COVID-19 and digitalization of congresses (6 questions) (see Supplementary 1).

The survey was available online for 35 days, from September 14 to October 19, 2020. Residents and young oncologists (less than 40 years old) were invited via e-mail, website, and social networks (except Twitter) through the National Association of Medical Oncology Residents (AERIO), the Society of Young Radiation Oncologists (SFJRO), and the National Association of Hematology Residents (AIH) to participate in this nationwide prospective survey. Two reminder e-mails were sent to increase the number of responses. The questionnaire was accessible from a computer, tablet, or smartphone using Google Forms.

### Statistical Analysis

Categorical variables are described as frequencies (percentage). Subgroup analysis was carried out according to hospital status (young resident, older resident, post-residency oncologist), medical specialty (radiation oncology, hematology-oncology, medical oncology), and online congress attendee since the beginning of COVID-19 pandemic (yes/no). The chi^2^ test was used for large samples (*n* ≥ 60) and Fisher’s exact test was used for small samples (*n* ≤ 60). For each test, statistical significance was set at a two-sided *p* value of < 0.05.

## Results

### Population Characteristics

One hundred eighty-three young oncologists answered the questionnaire, and 180 (98.4%) completed the whole questionnaire. Radiation oncologists were the most represented in this population (*n* = 93, 51.1%) followed by medical oncologists (*n* = 74, 40.6%). Hospital status was quite well balanced, with a predominance of older residents (second half of residency, *n* = 78, 43.3%) (Table 1).

**Table 1:** Population characteristics

Characteristics	Total population, *n* (%)
Total answers	183 (100%)
Hospital status	
Resident, first half of oncology residency (< 2.5 years)	50 (27.8%)
Resident, second half of oncology residency (> 2.5 years)	78 (43.3%)
Post-residency oncologist (< 40 years old)	52 (28.9%)
Specialty	
Hematologist-oncologists	15 (8.2%)
Radiation oncologist	93 (51.1%)
Medical oncologist	74 (40.6%)
Use of Twitter as a medical media	
Yes	51 (27.9%)
No	132 (72.1%)

**Fig. 1:**
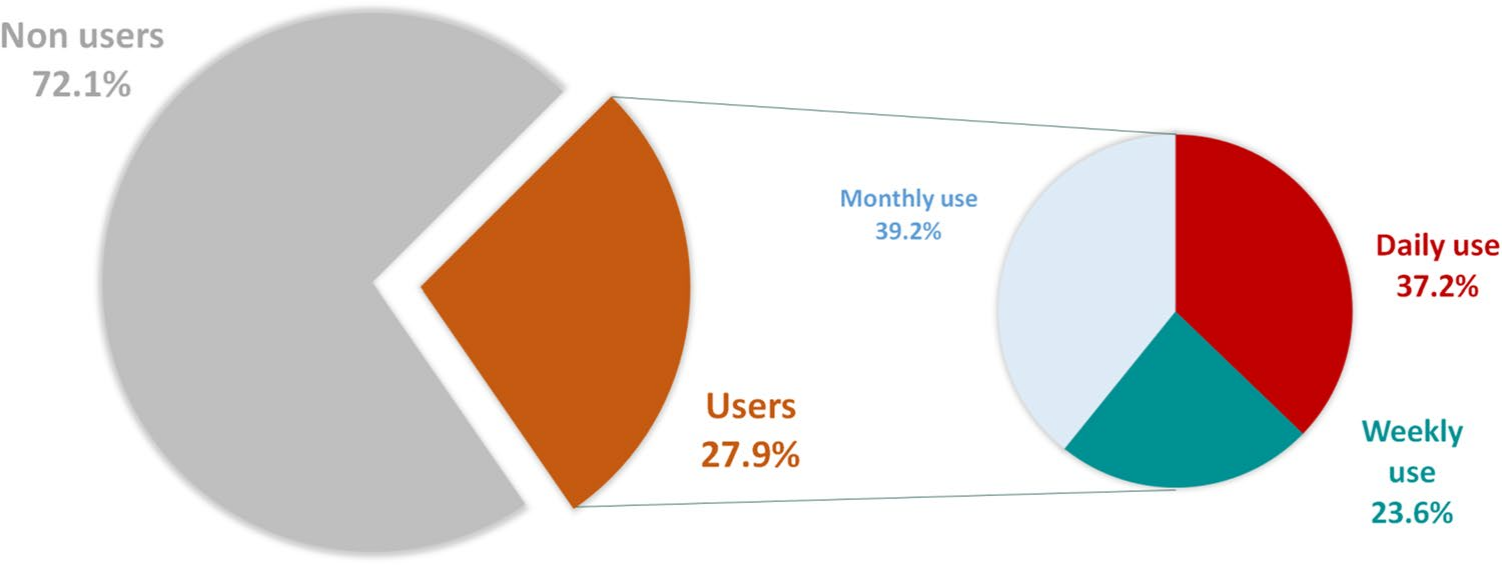
Profile of Twitter users among French young oncologists

Overall, 51 participants (27.9%) declare using Twitter as a medical media (see Fig. 1), 73.6% declare attending congresses, 70.9% attend academic courses (university degrees for example), and literature monitoring using dedicated mailing lists or websites is used by 50.5%. In contrast, 71 young oncologists (38.8%) own a Twitter account.

### Reasons Not to Use Twitter

**Fig. 2:**
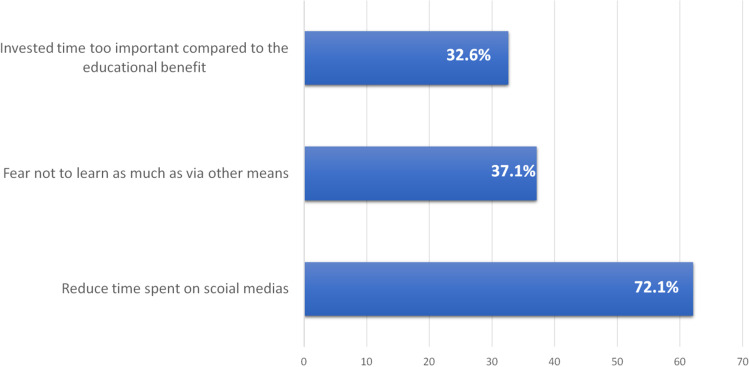
Main reasons not to use Twitter

Among young oncologists who declared not using Twitter as a medical media (*n* = 132, 72.1%), the main reason was to reduce time spent on social medias (*n* = 82, 62.1%), followed by the fear not to learn as much on Twitter as via other means (*n* = 49, 37.1%) or that the invested time would be too important compared to the educational benefice (*n* = 43, 32.6%, see Fig. 2). Eight participants answered that they had tried but had quit: in 6 of those 8 cases, the invested time was considered too important compared to the benefice.

### Benefits and Ways of Using Twitter

Fifty-one participants use Twitter as a tool for medical continuous education. Among them, 47 (92.2%) created their Twitter account during the residency and 4 out of 18 post-residency oncologists created their account after the residency.

Twitter users seemed to use it intensively, with a daily use by 19 participants (37.3%), and more than once per week in a majority of cases (*n* = 31, 60.8%, Fig. 1). Conversely, they tweet less than once per month in 76.5% (*n* = 39), whereas the remaining 23.5% (*n* = 12) only retweet medical influencers’ contents. Besides, they had few followers (< 50 followers in 80.4%, *n* = 41), compared to the number of accounts followed (> 50 accounts followed in 64.7%, *n* = 33, and > 100 accounts followed in 39.2%, *n* = 20).

All Twitter users followed world experts, 80.4% (*n* = 41) followed scientific societies, and 78.4% (*n* = 40) medical journals. Literature monitoring accounts (such as @OncoAlert) were used by 24 participants (47.1%). Interestingly, pharmaceutical laboratories play a much poorer role with only 13.1% of participants (*n* = 7) following such accounts. 82.4% of participants mainly followed English-speaking accounts.

Twitter users mainly spend their time on this media scrolling to read news (88.2%, *n* = 45), whereas 11.8% (*n* = 6) prefer exploring an account’s content, and the ideas and articles related to it. According to the users, Twitter’s main interest was to guarantee a fast and easy access to the opinion of world-class experts on medical news (74%, *n* = 37), more than the rapidity of access to medical information (22%, *n* = 11).

### Evolution of Twitter Use During the COVID-19 Pandemic

Among Twitter users, 41.2% (*n* = 21) estimated that they used more Twitter than 6 months before and 43.1% (*n* = 22) estimated that it was stable. In 29.4% (*n* = 15) of cases, congress virtualization due to COVID-19 pandemic pushed the user to use Twitter more than they used to. Live tweets are regarded as a necessary complement to oral presentations by 81% (*n* = 30) of Twitter users.

### Subgroup Analysis

We sought to determine whether population characteristics differed between Twitter users and non-users. A statistical difference could neither be found between these two groups regarding hospital status, medical specialty, or participation to an online congress (see Table 2) nor regarding the use of other means of continuous education (Table 3).

**Table 2:** Comparison of Twitter users and non-users

Characteristics	Twitter users, *n* (%)	Twitter non-users, *n* (%)	*p*
Total answers (*n* = 183)	51	132	
Hospital status			
Resident, first half of oncology residency (*n* = 50)	13 (25.5%)	37 (28.0%)	0.49
Resident, second half of oncology residency (*n* = 78)	20 (39.2%)	58 (43.9%)	
Post-residency oncologist (< 40 years old) (*n* = 52)	18 (35.3%)	34 (25.8%)	
Unknown (*n* = 3)	0 (0%)	3 (2.3%)	
Specialty			
Hematologist-oncologist (*n* = 15)	2 (3.9%)	13 (9.8%)	0.40
Radiation oncologist (*n* = 93)	28 (54.9%)	65 (49.2%)	
Medical oncologist (*n* = 74)	21 (41.2%)	53 (40.2%)	
Unknown (*n* = 1)	0 (0%)	1 (0.8%)	
Attended an online congress since the beginning of COVID-19 pandemic			
Yes (*n* = 84)	19 (37.3%)	65 (49.2%)	0.14
No (*n* = 99)	32 (62.7%)	67 (50.8%)

**Table 3:** Use of other means of continuous education depending on Twitter use

Other means of continuous education	Twitter users, *n* (%) 51 participants	Twitter non-users, *n* (%) 132 participants	*p*
Attending academic courses	41 (80.4%)	110 (83.3%)	0.64
Attending congresses	28 (54.9%)	82 (62.1%)	0.37
Literature monitoring using websites or mailing lists	27 (52.9%)	66 (50%)	0.72

## Discussion

To our knowledge, this work is the first to describe the characteristics of Twitter users but also non-users among young oncologists and the influence of COVID-19 pandemic on their Twitter activity. Our survey shows a very heterogeneous use of Twitter despite the large sample and its representativity of French young oncologist population. Nevertheless, these results are consistent with other studies [13].

On one hand, the majority of the survey participants do not have a Twitter account, mainly because of a defiance against overuse of social media in general and estimated insufficient benefits of the time spent on this platform. Indeed, time management is one of the major pitfalls when talking about social media (SoMe) [15].

On the other hand, close to a third of them use Twitter as a medical education tool and are clearly active: more than 40% connect every day. Twitter is for them a full part of their training, completing (and in some cases replacing) academic teaching. They prefer following expert accounts rather than journals or pharmaceutical companies. An explanation would be that they are willing to fully understand medical actuality through key opinion leaders, rather than just being informed. This need became stronger with the global pandemic and suspension of physical congresses. Students watching online talks alone are seeking for experts’ comments. In our study, more than 80% of them considered Twitter an essential complement to meetings, which is consistent with the rising use of Twitter during international congresses [16]. However, despite their intense presence on this media, only a few of French young oncologists express themselves otherwise than by retweeting experts’ posts. To remain feasible and maximize the number of responders, we did not explore in depth the reasons why they refrain from sharing their own views but we imagine that they might not feel qualified enough to express their opinion.

In our study, more than 40% of Twitter users increased their use of this social media since the beginning of COVID-19 pandemic. This underlines the challenge of digitalization of medical information, due to social distancing. Twitter plays an important role in such a process and can help users follow medical actuality when they cannot physically attend courses or congresses.

In this survey, we failed to identify significant differences between these two profiles that determine their use of medical Twitter. First, it should be noted that the goal of our questionnaire was to focus on Twitter use, more than on the characteristics of users. Moreover, reasons for using or not using Twitter seemed unrelated to medical specialty or hospital status as they are mainly related to personal beliefs. We can therefore assume such a difference does not exist. In both cases, residents lack guidance for a wise use of Twitter as a professional tool, some of them thus deciding to avoid it, the others to abstain posting original content. An American study already demonstrated that only few physicians, known as “superusers,” mastering the codes of their specialty and social media, are responsible for the majority of tweets in oncology [17].

Like Attai et al. [15] showed, SoMe can be tricky for physicians, especially young ones, having to manage simultaneously patient privacy, their own privacy, and building an e-reputation, without wasting valuable time. However, Twitter can provide many benefits to oncologists’ continuous education. Besides real-time and commented information, young oncologists could enlarge their network and access to virtual mentorship [18]. By contrast with our non-user responders’ opinion, Twitter can also help young oncologists to sort key papers. Indeed, tweets can predict most cited articles, leading to the development of altmetrics (non-traditional bibliometrics proposed as an alternative or complement to traditional citation impact metrics) [19]. For example in radiation oncology, it has been demonstrated that tweeting the link of a research article was correlated with the number of academic citations of this article [20]. Thanks to Twitter, young oncologists can also highlight their own work and participate in patient advocacy. Some institutions have even decided to include SoMe activities in academic advancement [21].

COVID-19 pandemic has underlined the importance of medical media in the medical education of French residents, which contrasts with the lack of formation they receive on their use. To help residents optimize their time on social media, some societies have written recommendations [22, 23]. These have multiple goals: protect physicians in training from blurred lines between personal and professional identities as well as organize their bibliographic monitoring, increase the altmetric of their articles, facilitate communication and mentorship. We believe such recommendations should be taught early in the medical education of young oncologists, in order to help them better understand this tool and to overcome their fears.

## Conclusion

In conclusion, close to a third of young French oncologists are using Twitter as a tool in order to learn mostly from key opinion leaders. This phenomenon increased with COVID-19 pandemic and the access to many online talks without experts’ live commentaries. However, guidance is needed to make the most from the time spent on this platform and provide a clearer perspective to a majority of physicians regarding the value of Twitter and potentially encourage its use in order to avoid the “superusers” bias.

## Supplementary Information

Below is the link to the electronic supplementary material.

Supplementary file1 (DOCX 13 KB)
