# Supplementary material for: Twitter as a Medical Media Among French Young Oncologists: Results from a National Survey
Source: J Cancer Educ. 2021 Nov 26;38(1):319–24. doi: 10.1007/s13187-021-02119-7 (PMC8626133; doi:10.1007/s13187-021-02119-7)
Supplement: Supplementary file 1 — Supplementary file1 (DOCX 13 KB) [file 13187_2021_2119_MOESM1_ESM.docx]

**Supplement 1. Survey questionnaire**

| **Questions** |
| --- |
| **Section 1: Medical curriculum**  Q1. What is your hospital status?  Q2. What is your medical specialty?  Q3. Which mean of initial medical formation have you used?*  Q4. Which mean of continuous medical education do you use?*  **Section 2: Twitter General Use**  Q5. Do you have a Twitter account?  Q6. Do you use Twitter as a medical media?  **Section 3: Characterization of Twitter non-users ****  Q7. Have you ever tried to use Twitter as a medical media?  Q8. If so, what reasons have pushed you to stop?*  Q9. If not, what are your hesitations regarding this network? *  Q10. Have you attended an online congress since the beginning of COVID-19 pandemic?  Q11. What do you suggest to replace post-presentation discussions?  **Section 4: Characterization of Twitter users*****  Q12. In which year did you create your Twitter account?  Q13. At what point of your curriculum have you created your Twitter account?  Q14. How often do you use Twitter?  Q15. What kind of account do you mostly follow? *  Q16. What is the main language of the accounts you follow?  Q17. How many accounts do you follow on Twitter?  Q18. How many followers do you have?  Q19. What is your common way of using Twitter?  Q20. According to you, what is Twitter’s main interest?  Q21. What kind of content do you share on Twitter?  **Section 5: Evolution of Twitter use due to COVID-19 pandemic and congress virtualization*****  Q22. How has your Twitter use evolved in the last 6 months?  Q23. Has congress virtualization due to COVID-19 pandemic pushed you to use more Twitter to follow them?  Q24. Have you attended an online congress since the beginning of COVID-19 pandemic?  Q25. If so, do you think that Twitter is complementary to oral presentations?  Q26. Do you think Twitter can replace post-presentation discussions during online congresses?  Q27. What other means do you suggest to replace post-presentation discussions in online congresses? |

***** Respondents were allowed to select more than one option (responses may not add up to 100%).

****** Section only available if no use of Twitter for medical purpose was declared.

*** Section only available if a Twitter use for medical purpose was declared.
